# A Habitable Earth and Carbon Neutrality: Mission and Challenges Facing Resources and the Environment in China—An Overview

**DOI:** 10.3390/ijerph20021045

**Published:** 2023-01-06

**Authors:** Min Zhang, Yan Qiu, Chunling Li, Tao Cui, Mingxing Yang, Jun Yan, Wu Yang

**Affiliations:** 1 Faculty of Resources and Environmental Engineering, Guizhou Institute of Technology, Guiyang 550003, China; 2 Engineering Research Center of Carbon Neutrality in Karst Areas, Ministry of Education, Guiyang 550003, China

**Keywords:** habitable Earth, environmental remediation, coal, carbon neutralization, China

## Abstract

Since the Industrial Revolution, the impacts of human activities have changed the global climate system, and climate warming has had rapid and widespread effects on the planet. At present, the world is experiencing a series of natural disasters, such as climate change, environmental pollution, biodiversity loss, and sea level rise, which pose a serious threat to the livability of the Earth. An international consensus has been reached that achieving carbon neutrality is the key to tackling climate change; it is also crucial to building a livable planet. To achieve carbon neutrality, energy is the main aspect, for which technology regarding resources and the environment is essential. In this context, we collected data, performed an in-depth analysis of the basic and structural characteristics of the development of the coal industry and environmental remediation, studied and judged the trends in regional economic development and demand growth, and closely examined the requirements of China’s development strategy, which focuses on the ideas of carbon peak and carbon neutralization in line with local development trends and economic system characteristics. We must build a livable Earth, promote the green and low-carbon transformation of regional energy, promote high-quality economic development, and ensure the safe supply of energy.

## 1. Introduction

**Figure 1:**
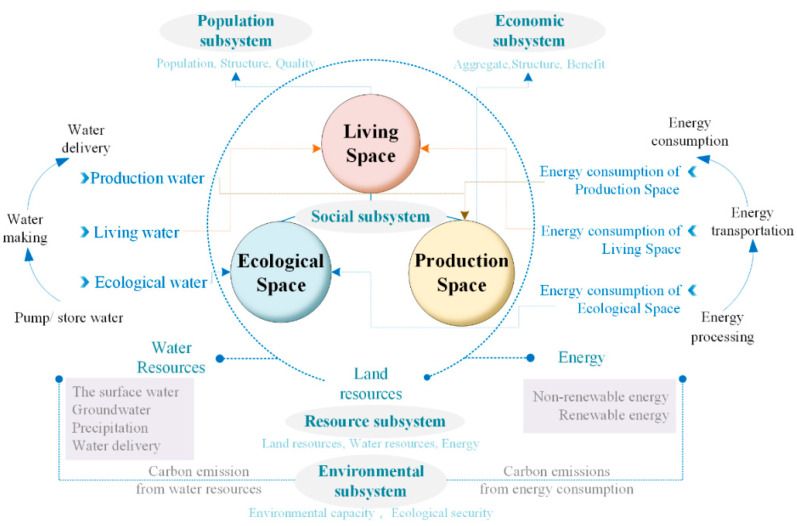
Habitable Earth: the systematic production–living–ecological space (Lin, G. [1]).

The fate of humankind depends on the livability of the Earth. As global energy and resource shortages, environmental pollution, climate change, natural disasters, and ecological damage continue to intensify, the Earth’s livability is facing unprecedented challenges. The ecological and environmental problems facing the world cannot be understood and solved by a single discipline, so we must train highly skilled and specialized people with a solid foundation to solve these problems not only today but also in the future. Carbon neutralization was proposed as an idea in response to the global greenhouse effect and climate change, with the aim of achieving climate neutralization and ensuring the livability of the Earth’s environmental system, which is one of the focuses of current geoscience. Therefore, carbon neutralization is closely related to geoscience. The current wave of scientific and technological revolution and industrial transformation is accelerating. New technologies are increasingly being applied in the field of natural resources. This not only provides new opportunities to address complex resource and environmental problems, such as energy transformation, achieving a circular economy, ecological environment deterioration, and urban space development and use, but also impacts traditional geological work theories, models, and methods. Therefore, we must accelerate interdisciplinary education and research, expand the research vision of traditional geoscience, and innovate within geoscience research and personnel training. The fundamental path to achieving carbon neutrality involves reducing emissions and increasing carbon sinks. The basic technological paths include zero-carbon, low-carbon, decarbonization, carbon compensation, and other technologies. Resources and the environment are indispensable for the zero-carbon new energy, energy storage, low-carbon energy conservation, energy efficiency improvement, new technology, creating circular economies, decarbonization of carbon capture, energy use, creating ecological carbon sinks, and solar radiation geoengineering, which can be expected in the future. Especially for CCUS and ecological carbon sinks, geoscience technology needs to play a leading role. Based on this context, in the form of a literature review, we analyzed the current situation regarding coal and environmental remediation, analyzed its drawbacks and shortcomings, and identified key scientific issues. We provide suggestions and directions for improvement and development, and our findings provide scientific support for the construction of an ecological civilization and natural resources management to achieve the implementation of major national strategies and solve key resource and environmental issues in the construction of major projects and to contribute to the innovative development and achievement of self-reliance through science and technology (Figure 1) [1,2,3,4].

**Figure 2:**
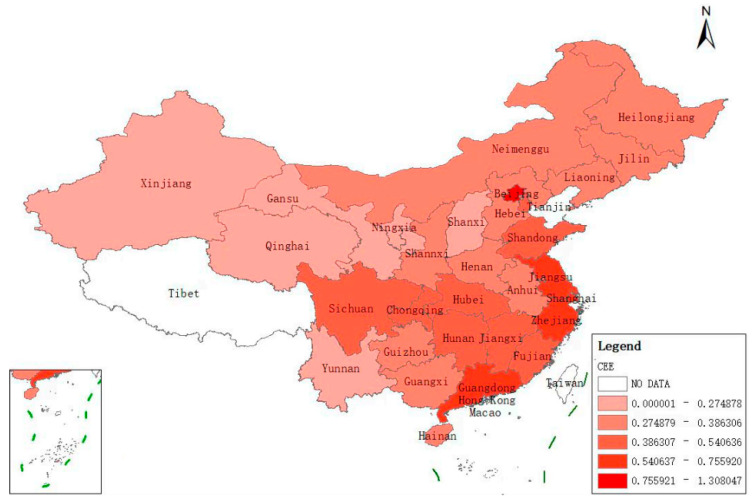
Average carbon emission efficiency (ACEE) in different Chinese provinces from 2016 to 2019 (Lin, G. [7]).

Achieving carbon neutrality provides opportunities and creates challenges for those involved in resources and the environment. Achieving carbon neutrality is a major change in the mode of economic and social development, which is currently difficult for most countries in the world. Energy in China has long been predominantly produced by coal. Of the three fossil fuels, coal, oil, and gas, coal burning emits more carbon than natural gas and much more than oil. As such, China is at a resource disadvantage compared with the United States of America and developed countries in Europe, where oil and natural gas account for a high proportion of the thermal power. Additionally, the scale of China’s industry is large, with nearly 70% of carbon dioxide emissions coming from industry, which is a much larger proportion than that of developed countries in Europe and the USA. At present, China’s economy and society are still in a stage of rapid development, with huge demands for space for urbanization, infrastructure construction, and improvement in people’s living standards, which all increase energy demand. This means that China’s carbon dioxide emissions will continue to grow both in total and per capita. However, the two carbon targets require a carbon peak in 2030 and carbon neutrality by 2060, with only 30 years between them. Time is tight and the task is enormous, necessitating strict requirements on the exploitation of resources and the environment (Figure 2) [5,6,7].

## 2. Literature Review of Low-Carbon Coal Exploitation and Use

**Figure 3:**
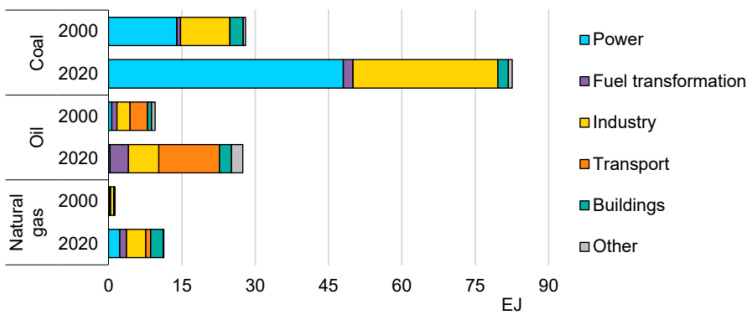
Fossil fuel consumption by sector in China.

In the coal industry, energy transformation is the most important aspect of achieving the dual carbon goals. China is a developing country, and with the further development of industry and urbanization, energy demand has steadily grown. Additionally, China’s coal-based resource endowments and energy supply structure provide an important guarantee for energy security, but they also pose challenges for the transformation to a low-carbon society. The main challenge in China’s energy transformation lies in the high proportion of coal in China’s energy resources and structure. The power shortages that have been occurring since the autumn of 2021 show that energy transformation is difficult to achieve quickly, and it needs to consider the available resources, energy security, and long-term emission reduction goals—the transformation must be completed step by step. Compared with the average world level, the proportion of coal in China’s primary energy consumption has been declining since the early 2010s, but still accounts for more than 50% of the total. Nearly 80% of China’s carbon dioxide emissions are produced from the consumption and use of coal energy, so the high-quality and low-carbon development of the coal industry is the key to achieving China’s goal of carbon neutrality. From strictly controlling the growth of coal consumption to gradually reducing the consumption of coal energy, it is essential to develop clean, low-carbon technologies and efficiently use coal to achieve the high-quality and low-carbon development of the coal industry, to accomplish the dual goals of energy security and carbon neutrality [8,9,10,11,12,13,14,15,16,17]. Here, clean, low-carbon technology and the efficient use of coal concern carbon emissions and emission reductions throughout the life cycle of coal production, combustion, conversion, chemical industry, and material use, which not only determine CO_2_ emissions, but also control atmospheric CH_4_ greenhouse gas emissions. Geological technology is an important part of the low-carbon technology system for the clean and efficient use of coal, in which resources and the environment play a vital role (Figure 3) [18].

### 2.1. Methane Emission Reduction Path and Key Technologies in Coal Mining

According to the IPCC2006 Guidelines, greenhouse gas emissions from the coal industry are mainly produced by the coal mining process, postmining activities, low-temperature oxidation, uncontrolled combustion, and abandoned coal mines. CO_2_ is the main greenhouse gas produced by low-temperature oxidation and uncontrolled combustion, and methane emissions are mainly produced by the coal mining process, postmining activities, and abandoned coal mines.

The emissions produced in the process of coal mining (including underground and open-pit mining) mainly refer to the emission of methane adsorbed in the coal bed caused by the disturbance of coal mining activities, which is released into the atmosphere in a free state. Methane emission during underground mining is discharged from the underground extraction and ventilation systems, some of which can be recycled. Emissions from postmining activities mainly refer to those produced by coal sorting, storage, transportation, and crushing before combustion. In the process, methane is produced by the slow release of residual gas in coal. The emissions from abandoned coal mines mainly include the methane produced by the slow release of residual gas in coal mines from surface fissures or human-made channels after coal mining stops. China is the largest coal producer in the world, with output accounting for 50% of the world’s total. Underground mining is the main method of coal mining, and methane emission from underground coal mining is the main source of methane emissions in China. The methane produced from postmining activities has also become one of the main sources of coal mine methane emissions in the country [19,20].

#### 2.1.1. Methane Emitted from Coal Mines and Its Characteristics

Ma [21] calculated the CH_4_ emissions from coal mining and postmining activities in China from 2010 to 2016 using the national standard emission factor method for the key emission sources of coal emissions from underground coal mining and postmining activities, and using the default emission factor method for other emission sources. From 2010 to 2016, the CH_4_ emissions from coal mining and postmining activities in China increased first and then decreased, and the largest emissions were from underground mining. In 2010, fugitive CH_4_ emissions totaled 25.25 million tons. Considering the pressure on resources and the environment, China has devoted many efforts toward adjusting the energy structure and reducing the proportion of coal consumption since 2011, thus forcing coal production to reach its peak in 2013; accordingly, fugitive CH_4_ emissions reached a peak of 27.16 million tons. After that, it decreased to 22.69 million tons in 2016. During this period, the emissions from underground mining, postmining activities, open-pit mining, and abandoned mines accounted for 83%, 13%, 3%, and 1% of the total emissions, respectively. Due to the high gas content in underground coal mines and the low level of integration of mining and ore dressing, the proportion of emissions from underground mining and postmining activities in China is relatively high compared with that of other major coal-producing countries. From 2010 to 2016, the amount of CH_4_ recovery and use from coal mining and postmining activities in China increased each year; the peak net CH_4_ emissions, after deducting the amount of recovery and use, moved forward to 2011. Mine gas is the main hazard in the process of coal mine production. In addition, the CH_4_ in the gas is a high-quality clean energy, so is a highly valuable resource. Therefore, the importance of CH_4_ recycling is increasing. From 2010 to 2016, CH_4_ recycling annually increased by 17.0%, and the proportion of recycling in total emissions increased from 9.4% to 26.6%. The actual net CH_4_ emissions to the atmosphere, after deduction of the amount recovered, were further reduced.

**Figure 4:**
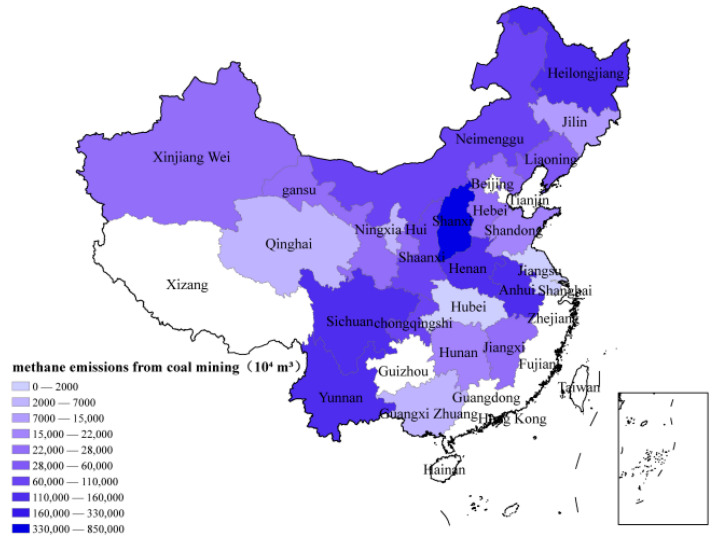
CH_4_ emissions of coal mining industry in various provinces (Zhu, A. 2022 [28]).

China’s methane gas from coal mines also has distinct characteristics. First, the concentration of methane gas in coal mines widely varies. During coal mining activities, the volume fraction of methane in drainage gas, ventilation gas, and surface coal bed methane in goafs is 0.5–95%. This wide range of the methane volume fraction increases the difficulty of reducing methane emissions. Second, low-concentration methane accounts for a large proportion of the methane in coal mines. Due to the complex geological conditions of coal in China, including the low permeability of coal seams, most mines are located in “three soft” coal seams, which are not conducive to drilling. Therefore, low-concentration methane, with a volume fraction of 8~30% in the extracted gas, accounts for a large proportion of the total coal mine gas, which is not easy to use, and is also the main reason for the low use rate of methane emissions from coal mines. Third, the ventilation gas is thin but accounts for the largest amount of emissions. To ensure the safety of underground miners, the upper limit of the gas volume fraction is set in key parts of the underground ventilation. To ensure the health and safety of underground workers and safe production, the ventilation rate must meet the relevant requirements, resulting in a huge amount of gas being directly discharged into the air. However, under the existing technical conditions, effectively using such low-concentration ventilation gas is difficult (Figure 4) [22,23,24,25,26,27,28].

#### 2.1.2. Coal Mine Methane Emission Reduction Pathways

At present, many emission reduction technologies are applied in coal mines, which follow the overall path of coordinated technology, economy, and policy development under the guidance of the emission reduction principles of green development, with overall coordination, applying multiple measures, and with support and guarantee. The specific path mainly includes six aspects. The first is governance at the source. The aim is to vigorously promote the application of precise coal mine gas drainage technologies such as intelligent drainage, break through technical bottlenecks such as soft coal seam hole collapse and abandoned coal mine gas development, increase the volume fraction of gas drainage, and reduce coal mine gas emissions from the source. The second aspect is technical support. Technical and economically feasible breakthroughs will be achieved in key technologies of coal mine gas use, use costs will be reduced, and technical support and guarantees will be provided for coal mine gas emission reduction. The third is quality use. Given the different volume fractions in coal mine gas and combined with the various technical conditions of coal mine gas use, the diversified, comprehensive use of civil gas, industrial boilers, coal mine gas power generation, gas purification and use, oxidation, and heat supply will be attained. Fourth is policy guarantees. The aim here is to introduce and improve a variety of policies to reward and support regional differentiation, such as financial subsidies, tax and fee relief, and price increases for power generation, and to explore commercial operation modes for effective cooperation between private and state-owned enterprises. The fifth aspect involves monitoring and accounting. Relevant departments and enterprises are encouraged to conduct research on coal mine gas monitoring technology and accounting methods. The orderly construction of pilot projects for coal mine gas monitoring will be promoted, giving full play to the leading role of demonstration projects. Sixth is carbon market improvements. Combined with the market development stage, orderly carbon emission reductions through coal mine gas use will be promoted in the carbon trading market, and project income will be increased through carbon emission reduction income, which should stimulate the enthusiasm of enterprises to use coal mine gas [29,30,31].

#### 2.1.3. Key Technology of Methane Emission Reduction in Coal Mines

At present, three key technologies are applied for coal mine methane emission reduction:

(1) High-concentration gas emission reduction technology. Coal mine gas power generation, civil gas, and automobile fuel are the main uses of high-concentration gas (generally considered as having a volume fraction ≥30%). This technology is mature and can produce higher economic benefits, having become the most valuable gas use technology to promote coal mine methane emission reductions in China. As an extension of the use mode, low-concentration gas compression and purification liquefaction is another methane emission reduction method that shows emission reduction potential.
(2) Low-concentration gas emission reduction technology. For low-concentration gas (generally refers to a volume fraction of 5–30%), the main method to reduce emissions is power generation. Because low-concentration gas is explosive in the 5% to 16% range, the occurrence of gas explosion accidents must be prevented during transportation and use. After the safety problems with low-concentration gas transportation technology were solved, low-concentration gas power-generation technology has gradually matured through the accumulation of experience in the process of large-scale application. The key difficulty with low-concentration gas purification technology is economically and efficiently separating CH_4_/N_2_. At present, the commonly used technology is pressure swing adsorption.
(3) Ventilation gas emission reduction technology. The volume fraction in ventilation gas is generally below 0.75%, which is characterized by high emissions, low concentration, and difficult use. Effective ventilation gas use technology requires breakthroughs in the process of methane emission control in China’s coal mines. Ventilation gas worldwide is used in two ways: as the main fuel, using counterflow thermal oxidation and counterflow catalytic oxidation technology, and as auxiliary fuel, using hybrid combustion technology. At present, one of the promising technologies is bidirectional regenerative oxidation. The technology consists of an electric heating unit, a bed body (generally heat storage ceramics), and a heat exchange unit in the center. In the initial stage, the middle part of the bed is preheated to the temperature of methane self-ignition (1000 °C) by electric heating. The complete process cycle includes two changes in the direction of gas flow, so half a cycle is required to change the direction of the gas flow once. During the first half of the cycle, the ventilation air flows from one end of the reactor at ambient temperature and passes through the reactor. When the temperature of the mixed gas exceeds the self-ignition temperature of methane, the oxidation reaction of methane occurs near the center of the bed. The heat generated by combustion and the unburned gas continue to pass through the bed body and transfer the heat to the part far from the center of the bed body. When the far bed is sufficiently heated, the near bed drops in temperature due to the new gas entering at ambient temperature. To continue the reaction, the system uses an automatic control system of the inlet and exhaust valves to reverse the direction of the air flow in the reactor to enter the second half of the cycle. Fresh air enters from a distance and absorbs heat from the bed, and the methane near the center of the reactor reaches the self-ignition temperature, oxidizes, and releases heat, which is transferred to the bed near the reactor and then discharged. As such, the central area of the bed body is provided with a high-temperature area with a fixed width, and the width is basically kept constant. The central temperature plus the adiabatic temperature rise can reach 1000 °C, and then the heat is transferred out through the heat exchanger and used. At present, many oxidation heating projects have been implemented in China.

### 2.2. CCUS and Carbon Dioxide Removal from Coal

As a emission reduction technology, carbon capture and storage (CCS) is important for China to implement its low-carbon development strategy and achieve green development. To advance CCS technology and increase its scale, huge capital and operating costs and additional energy consumption will be required, and some problems with CCS regarding safety and integration with large-scale demonstration projects must be solved. Combined with China’s national conditions, CO_2_ use has been added to demonstration projects on the basis of the original three aspects of CCS: carbon capture, use, and storage (CCUS). The main CCS methods include enhanced oil recovery (EOR), enhanced coal bed methane recovery (ECBM), food-grade CO_2_ refining, and other industrial use methods.

In 2020, CO_2_ emissions from coal energy consumption accounted for more than 70% of CO_2_ emissions from fossil energy activities in China, thus being the main form of carbon emissions in China. The useful elements in fossil energy are mainly carbon and hydrogen, and coal energy is characterized by high amounts of carbon. Regardless of the combustion or conversion technology used, to achieve low-carbon use of coal energy, that is, low-emission use, the extra carbon must be used. CCUS, as the main decarbonization technology, is the only technology that not only directly reduces carbon emissions in key areas, but also reduces the existing atmospheric CO_2_ concentration to neutralize unavoidable carbon emissions. In China, reforming the energy structure and substituting energy based on fossil energy will take time. CCUS technology is key in reducing CO_2_ emissions from fossil energy, especially coal, consumption and use represented, which will determine the process and direction of low-carbon development of the coal industry to a certain extent (Table 1).

#### 2.2.1. CCUS Trends and Objectives

**Figure 5:**
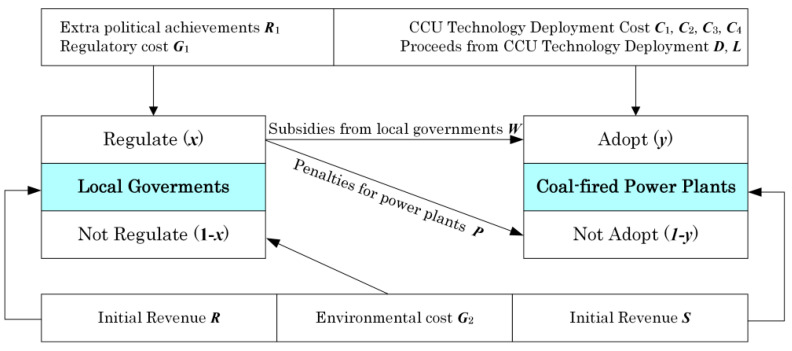
Behavioral strategy framework considering local governments and power plants (Nie [36]).

China has a huge potential market for CCUS applications. The total primary energy production is estimated to reach 4.3 billion tons of standard coal in 2030, and the carbon dioxide emissions will reach a peak of 11.2 billion tons. In terms of storage and application, taking EOR as an example, approximately 13 billion tons of crude oil geological reserves in China are suitable for EOR, which can improve crude oil recovery by 15%, increase production reserves by 1.92 billion tons, and store about 4.7–5.5 billion tons of carbon dioxide [30]. The applications of CCUS technology mainly include physical, chemical engineering, and biological applications. The physical applications mainly include, among others, beer and carbonated beverages; oil displacement agents for tertiary recovery of petroleum; inert gas shielded welding in welding process; cold energy of liquid and solid CO_2_ for refrigeration, storage, and transportation of food and vegetables; natural oxygen reduction in fruits and vegetables; modified atmosphere preservative; and supercritical CO_2_ extraction. The chemical applications mainly include inorganic and organic fine chemicals, polymer materials, etc. For example, CO_2_ can be used as a raw material to synthesize urea and produce light, nano-grade, ultra-fine active carbonate; CO_2_ is catalytically hydrogenated to produce methanol; CO_2_ is used as a raw material to synthesize a series of organic raw materials; CO_2_ and epoxide are copolymerized to produce high polymers; and a series of hydroxylated carbon chemicals was developed by converting CO_2_ into CO. The biological applications mainly include the use of microalgae to fix CO_2_ and convert it into biofuels and chemicals. After the initial demonstration stage, the cost of biofertilizers, food, and feed additives is expected to decrease by 10–20% when the production capacity of CCUS is doubled [31,32,33,34,35]. Nie [36] established an evolutionary game model to analyze the interaction process and evolution direction between local governments and coal-fired power plants. The findings showed that when the unit price of hydrogen drops to 15.9 RMB/kg or the unit price of carbon dioxide derivatives rises to 3.4 RMB/kg, the evolution stability strategy of the system changes, and the power plant turns to CCU technology (Figure 5).

#### 2.2.2. Application and Effect of CCUS

**Figure 6:**
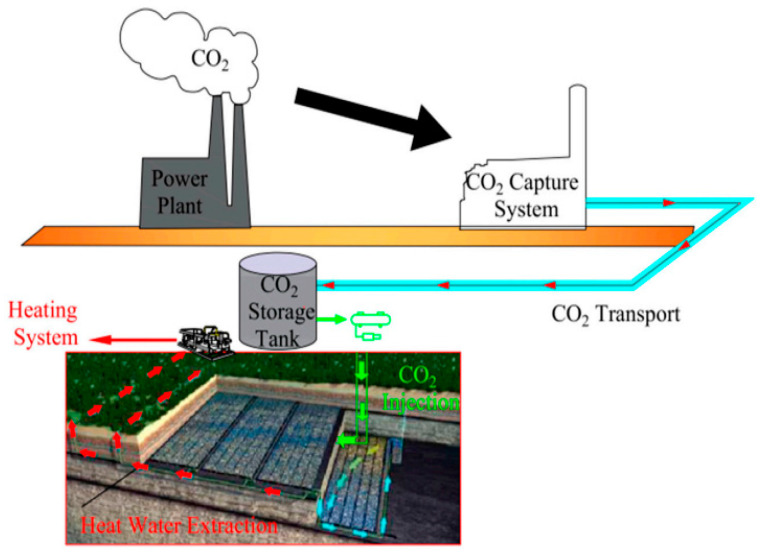
CO_2_ sequestration in coal mines (Wang [39]).

(1) Coal-fired power plants CCUS: Coal-fired power plants are the main consumers of coal in China. In 2020, CO_2_ emissions from coal-fired power plants accounted for more than 30% of the total carbon emissions, constituting the largest industrial fixed point source of CO_2_ emissions in China. Coal-fired power plants that apply CCUS can reduce their carbon emissions by 90%, thus making coal-fired power generation a relatively low-carbon power generation technology. However, this technology, both in China and abroad, is still in the stage of industrial demonstration. The scale of domestic coal-fired power plant flue gas CCUS demonstration projects is small overall, and no industrial operation precedent exists for large-scale (scale ≥ 1 million tons/year) demonstration projects. Only two projects have been implemented abroad: the Canadian Boundary Dam Million Tons CO_2_ CCS Project and the American Petra Nova 1.6 Million Tons CCS Project. The technical CCUS process of coal-fired power plants includes many technical links such as flue gas CO_2_ capture, transportation, use, and storage. At present, progress has been achieved in many fields of technology research and development and in engineering application explorations, and CCUS is expected to achieve commercial-scale deployment in the near future. Research is still required on methods of CO_2_ storage capacity evaluation and site selection exploration, source–sink matching optimization of geological storage, CO_2_ injectability, storage mechanisms, storage stability and safety, reservoir fluids (CO_2_, oil, gas, water, etc.). The effectiveness, safety, economic theory, and technology of CCUS, including the percolation and physical and chemical reaction mechanisms, have rapidly developed. The requirements for the commercial deployment of efficient CO_2_ geological storage technology, efficient oil and gas displacement technology, geological storage safety assessment and monitoring, and early warnings have been preliminarily met. CCUS for coal-fired power plants in China started relatively late, and the geological conditions of CO_2_ storage are relatively complex, so its technology research and development and demonstration projects are relatively lagging [37,38].
(2) Coal chemical industry CCUS: The CO_2_ emissions of the coal chemical industry rank first among the three major energy and chemical industrial chains (coal chemical, petrochemical, and gas chemical industries) in China. The CO_2_ emitted by the coal chemical industry in China was estimated to be approximately 400 million tons in 2020, which is a fixed point source of CO_2_ that cannot be ignored. The CO_2_ emissions of the coal chemical industry are characterized by high intensity and large scale from single emission sources and a high concentration of CO_2_ emitted by the production process. These low-cost, high-concentration, and large-scale CO_2_ sources result in the levelized cost of CCUS projects in the coal chemical industry being lower than in other industries such as thermal power, iron, steel, etc. This is the special advantage of CCUS technology implementation in the coal chemical industry. This is also the first opportunity for China to implement low-cost and large-scale CCUS demonstration projects. Therefore, incorporating CCUS into the coal chemical industry has also been listed as a priority action for the implementation and large-scale deployment of CCUS demonstration projects in China. At present, only three demonstration projects of the coal chemical industry and CCUS have been constructed in China: the 100,000 tons/year CO_2_ saline water storage project of Ordos Coal Chemical Industry (coal to oil) of the State Energy Group, the 50,000 tons/year CO_2_-EOR demonstration project of the Northern Shaanxi Coal Chemical Industry (coal to gas) of Yanchang Petroleum, and the 50,000 tons/year CO_2_-EOR demonstration project (coal to methanol) in the Changqing Oilfield. The projects demonstrating CCUS in the coal chemical industry that have been implemented in China are small in scale, narrow in implementation objectives, relatively single in technical methods, and have not yet been applied in the fields of synthetic ammonia, coal-to-olefin, or coal-to-aromatics, so demonstration projects of the coal chemical industry with CCUS with a capacity of one million tons are urgently required. The technical and engineering capabilities of large-scale industrial demonstration projects need to be improved. In 2021, to promote the high-quality development of a modern coal chemical industry and the safe and efficient use of clean and low-carbon energy, the Ningdong Energy and Chemical Industry Base began to build a million-ton CCUS demonstration project. Moreover, China Petroleum and Chemical Corporation has built a 1 million tons/year CCUS (coal gas tail gas CO_2_ capture + EOR) in the Qilu Petrochemical-Shengli Oilfield. This demonstration project is the first million-ton CCUS project and the largest full-process CCUS project in China (Figure 6) [39,40,41,42,43,44,45,46,47,48].
(3) Large-scale coal bases have formed concentrated areas for the development and use of coal resources in China, which will help the state to enhance their macro-control over coal resources, to adjust and optimize the coal industrial structure, and to strengthen the comprehensive use of coal resources. In terms of geographical space, coal power, co-generation, the coal chemical industry, and other high-energy-consumption and high-emission enterprises are concentrated in large coal bases and in northwest China. These large coal bases emit large amounts of CO_2_ and have high carbon emissions per capita and per unit output value; here, achieving carbon emission reductions is arduous. However, many CO_2_ emission sources are concentrated in coal bases, which provide the basic conditions for the cluster-scale deployment of CCUS. Additionally, CCUS is the only technical path available for large coal bases to achieve near-zero CO_2_ emissions. CCUS and large coal bases have a high degree of source–sink matching. The potential for CO_2_ geological sequestration in China is huge, and the areas favorable for CO_2_ sequestration are the Bohai Bay Basin, Songliao Basin, Ordos Basin, Junggar Basin, Tarim Basin, and Sichuan Basin, which are highly geographically coincident with the CO_2_ emission sources of large coal bases. Through cluster-scale CCUS deployment, the scale and agglomeration effects of coal bases for CCUS can be achieved, the cost of regional CO_2_ transmission and pipeline network construction can be reduced, and large-scale and relatively low-cost CCUS projects can be realized. Using CCUS at coal bases can improve the rate of use of energy resources developments. The Ordos Basin, Junggar Basin, Tarim Basin, and other CO_2_ basins are rich in coal, oil, gas, and groundwater resources, and thus are appropriate for CO_2_ storage and use in CO_2_-Eor, CO_2_-ECBM, saline aquifers, etc., to realize the comprehensive development and use of energy resources. For example, the implementation of CO_2_-ESWR in Xinjiang, Inner Mongolia, and other western regions will help to alleviate the key problems caused by CO_2_ emissions and water demand in coal bases. CO_2_ sequestration in coal goafs also has considerable potential for carbon emission reduction, which can improve the extraction rate of coal mine gas and control the atmospheric emissions and leakage of coal mine gas [48,49,50,51,52].

### 2.3. Green Coal Mining

Green mining refers to the strict implementation of scientific and orderly mining in the whole process of mineral resources development. Additionally, with green mining, disturbances of the mining area and its surrounding environment should be controlled within the scope of natural regulation. The feasibility of scientific mining and the controllability of environmental impact in the whole life cycle of mineral resources development are emphasized [53,54].

**Figure 7:**
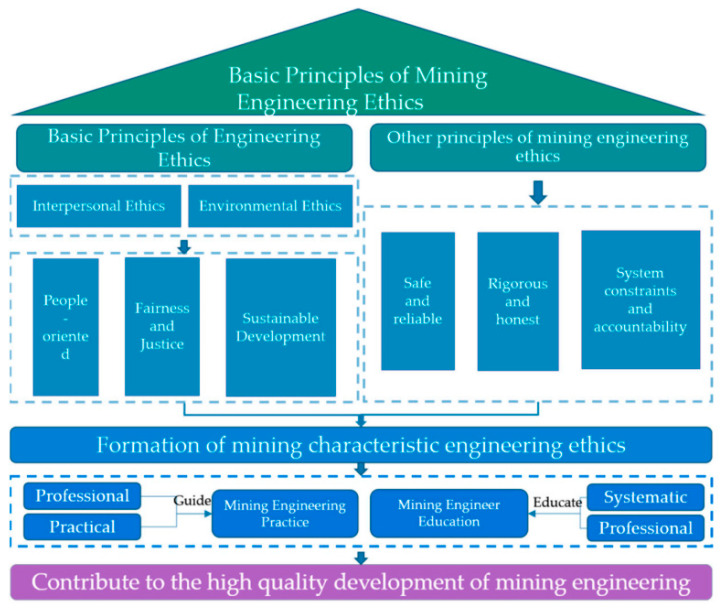
Basic principles of mining engineering ethics (Wang, F [54]).

Lv Xinqian [55] stated that the basic connotation of green mines entails legality, low consumption, environmental protection, recyclability, and innovation. Green mining provides a new idea for mine management and development planning; it allows not only for the improvement in the ecological environment of the mine, but also for the scientific and rational planning and development of mineral resources, improvements in the use rate of mineral resources, the reduction in waste of resources, and the promotion of the healthy development of the mine economy and the finding of a sustainable development path. Huang Jingjun [56] reported that green mines minimize the waste and consumption of mine resources on the basis of maximizing the protection of the ecological environment to seek sustainable economic development. The ecological development concept of green mines applies to the whole life cycle of mine development, including mine surveys, planning and design, construction, development, closure, and beyond. This complete process can fully take advantage of green mining methods in each link. Accurate implementation is required to effectively control the whole ecological process, maximize the use of mineral resources, and reasonably assess the ecological environment. Wang Yunjia [57] found that green mining does not produce a green product, but the rational planning and deployment of resources in mining areas based on the overall, global, and systematic consideration of various factors are considered green, which involves the coordination of various parts, maximizes the overall effect, and forms a network to realize the comprehensive, three-dimensional, and coordinated development and use of mining resources. The scope of green mining includes the whole process of mine activities, from geological exploration, mine design and construction, mining, smelting, and processing, as well as the restoration and reconstruction of the ecological environment after the closure of the mine. With a scientific and prudent attitude, mineral resources should be rationally exploited in a safe and environmentally friendly way on the premise of low consumption and high efficiency. Internal and external costs should be reduced overall under the constraints of recycling and the low-carbon economy, so as to take full advantage of human resources and precisely plan every link in the whole mining chain. This is required to sustainably develop mine resources and to protect the ecological environment (Figure 7) [58,59,60,61].

#### 2.3.1. Theoretical Basis of Green Coal Mining

China has promulgated the Basic Conditions for National Green Mines regarding building green mines, which stipulates aspects of standardized management, comprehensive use, technological innovation, energy saving, emission reduction, environmental protection, and land reclamation. The document stipulates aspects of the construction of green mines, but these guidelines have some problems, such as imperfect content indicators, low quantification of assessment indicators, low weight of forward-looking and environmental protection indicators, and not fully considering the distribution characteristics and industrial structure characteristics of open-pit coal mines in China, which make them lag behind the actual situation. This document cannot play an effective role in guiding the construction of green open-pit coal mines. Three main theories support the construction of green coal mines [61,62,63]:

(1) Green economy theory: Green economy is a new economic form based on traditional industrial economy and aimed at promoting the harmony between the economy and the environment. The aims of green economy are to change and eliminate the factors that are unfavorable to the environment and human beings in the process of production, circulation, distribution, and consumption in the traditional industrial economy, and to achieve economic growth in the process of industrial development. A green economy is not only manifested in a healthy environment and comfortable living during production, construction, and life, but also in the harmonious coexistence between human beings and nature. It also requires the coordinated development of social–economic–ecological systems, which requires the coupling and supplementing of sustainable development and circular economies [63,64,65].
(2) Precision mining theory: Yuan Liang [66,67,68,69] introduced the scientific concept of precise coal mining in consideration of the numerous challenges faced by coal mining in the new era and the new scientific and technological advances. He stated that precise coal mining is based on transparent space geophysics and multi-physical field coupling, supported by intelligent perception, intelligent control, the Internet of Things, big data, cloud computing, etc. Precise coal mining also involves risk identification, monitoring, and early warning. It is a new method used to accurately, safely, reliably, intelligently, and precisely mine in time and space with fewer people. Yuan Liang also introduced the concept of green resources during the construction of green mines. Green coal resources refer to the coal resources that can meet requirements regarding coal safety, technology, economy, and the environment, and support the scientific increase in coal production capacity and development. Precision mining theory is the extension of green mining theory, which combines the connotation of green mining theory, the requirements of a new round of scientific and technological innovation such as Internet+, and intellectualization in the new century, and indicates the direction for coal resources development.
(3) Fuzzy theory: In coal mining, objects are sometimes hard to describe with a deterministic mathematical value, given their uncertainty, which complicates the expression of some object information. The concept of fuzzy theory originated from the Department of Electrical Engineering at the University of California, Berkeley. Fuzzy theory involves fuzzy set theory, fuzzy reasoning and control, and fuzzy logic theory. The fuzzy concept provides mathematical support for the description of the uncertainty inherent in nature and human society. So far, the extensional fuzzy system has been scientifically described, which is the standard type 1 fuzzy set. Fuzzy theory was first proposed by Russell in the 1920s. In his research on mathematical problems in this field, he found that the description of some problems were characterized by “ambiguity”. These “ambiguities” were not accurate in expression, but they could contain some guiding information on the problem, such as language that enabled the recipient of the question to understand the meaning of the question to a certain extent, such as “how much”, “old or new”, and so on. In 1965, based on the above discoveries, Zadeh continued to study this problem, and published some papers clarifying that the membership function can be used to describe the degree of fuzziness of objects, so the solution of the problem of fuzziness of things was no longer confined to the set theory introduced by Cantor. The membership function can be used to calculate the fuzzy problem, which lays the foundation of the theory [70,71,72,73].

#### 2.3.2. Green Mining Technology System

While recovering resources, mining seriously impacts the environment. These impacts are mainly on the surrounding land environment, atmospheric environment, water resources, and so on. As such, reducing the occupation of land resources and rapidly reclaiming land in open-pit coal mines have become factors limiting their green development.

(1) Integration of mining, drainage, and reclamation: The engineering of open-pit mines mainly involves the mining, stripping, and transportation of ore and rock and the disposal of stripped materials, which destroys a large amount of land every year and is increasing at an annual rate of 8~9%. The traditional reclamation technology used for open-pit mines is mostly applied after the mining of the whole area is completed and the pit is closed. The aim of the integration of mining, drainage, and reclamation technology is to change the idea of first destroying and then restoring, and to pay attention to the timeliness of reclamation work so that strip mining and reclamation are simultaneously performed. Reclamation can be performed while dumping; the exposure time of the dump can be reduced, as can water and soil loss; and the effects of the overburden and pollution of land resources caused by open-pit mining can be mitigated to a large extent. The key problem in the integration of mining, dumping, and reclamation technology is coordinating the progress and space–time relationship of open-pit mining, dumping, and reclamation according to the local climate conditions and actual situation, and to ensure the appropriate storage and use of topsoil.
(2) The comprehensive control of dust during open-pit coal mining: The open-pit coal mine area can be divided into disturbed and undisturbed areas according to the location of personnel and equipment for operation. The main object characterizing the undisturbed areas is the waste dump, which may be located in a reclaimed area or a covered area according to the reclamation process. This covered area is an area of concentrated dust production in the undisturbed area, and the dust is natural dust. Disturbed areas are where the production occurs, including perforation, blasting, mining, loading, transportation, and discharge. The disturbances in this area produce a large amount of dust, with variable dust sources; the type of dust is disturbed dust. According to the characteristics of different dust types, the dust control and reduction methods differ. The main technical means of managing dust in undisturbed areas is the spraying of dust suppressant, which attaches the dust particles to the crust of the non-disturbed area. Dust weight is increased to improve the wind load resistance.
(3) Water conservation technology for open-pit coal mines: The protection and recovery of water resources in open-pit coal mines are major environmental concerns with open-pit coal mining and important factors restricting their large-scale development. Open-pit mines produce the funnel effect, which seriously damages the surrounding groundwater resources, which are difficult to recover. The fundamental reason for this is that the water-resisting layer above the coal seam is stripped by blasting, so the water-resisting layer is missing. The structure of the inner material is loose, and the permeability coefficient is large, so water movement is not resisted or blocked. Open-pit water conservation mining mainly includes the following aspects:
  (i) Key technologies and methods for the reconstruction of aquifers and aquicludes of waste dumps;
  (ii) Reuse of solid mine waste and selection of aquiclude material.

#### 2.3.3. Smart Mines

Intelligent mining is a comprehensive system that digitizes various types of static and dynamic information involved in the engineering activities required for mining mineral resources on the mine surface and below ground. This information is managed by a computer network, and space technology and real-time automatic positioning and navigation technology are used to remotely and automatically control operation and mining during the mine production process. Intelligent mining is characterized by deep interconnection, high sharing, intelligent service, and visual display. From the perspective of coal mining enterprises, the penetration of high-tech systems such as artificial intelligence, the Internet of Things, and cloud computing into the automation, mechanization, and informatization of coal mining enterprises is the basic definition of intelligent mining.

Accurate positioning systems: In the process of coal mining, to ensure that coal mining can be performed step-by-step according to the plan, accurate positioning is required. However, no complex electromagnetic signal can exist in a mine and satellite signals cannot be received, so the difficulty of positioning and navigation is increased. At present, positioning information systems based on GIS are some of the most widely used positioning systems in China, which include five core technologies: high-precision navigation suitable for complex magnetic field environments, local positioning and navigation chips, underground high-speed wireless communication, underground high-precision positioning, and underground navigation avoidance. The application of these five technologies can increase the accuracy of underground positioning systems and further guarantee the smooth operation of coal mines. The humid and complex electromagnetic environments of underground coal mines seriously restrict the application of detection technology, so underground environment detection technology has currently become important for underground status analysis in China’s coal mining industry. Identification technologies such as environmental detection and vibration detection have become the core of underground environmental perception in China’s coal mines, which mainly include four aspects: high-precision penetration technology, coal detection sensors, mining equipment sensors, and specific physical environment detectors. These four technologies are inter-related and work together, and can more accurately transmit complex and extreme underground environmental information to the surface, and enable direct detection during coal mining.

Data analysis systems: Data analysis is key in intelligent coal mining. Because intelligent coal mining integrates the operation of eight subplatforms, data analysis is crucial both from technical and practical operation aspects. The logic module of digital coal mine systems needs to obtain actual data from many sensors; through the effective analysis of these data, the relevant laws of coal mining are determined and successfully applied to the control of intelligent mining. Accurate data analysis systems can help people more clearly understand the problems and bottlenecks encountered in coal mining and solve them. Although video surveillance technology has the support of related technology and equipment, the administrator must understand the real-time situation of underground operations. The video monitoring of underground coal mines is achieved through the video monitoring system of a command center and is used to observe changes in coal seam inclination and the operation of underground mining equipment in real time. Operators can know the specific underground work situation in real time through the display, which can help with avoiding problems in the production process. Video surveillance technology can help workers to further understand the coal mining, as can the information obtained from various real-time sensors. The application of video surveillance technology can also solve the problems experienced by intelligent coal mine systems through manual intervention, which further ensures the smooth process of coal mining.

Underground robot technology: The geographical environment of mines in most areas of China is relatively complex, which poses various safety hazards to mine workers to a certain extent. As such, at this stage, China has developed underground robots to address the technical problems in coal mines. Underground robots can replace human workers to complete the needed operations, which further facilitates underground operations. However, at this stage, some problems need to be solved, such as high power consumption, inability to self-repair, and so on.

## 3. Literature Review of Environmental Restoration and Governance

China is one of the countries with rich mineral resources, where more than 190 kinds of minerals have been developed and used. However at the same time, this development has come at the cost of environmental problems due to mining, but studies of the mine environment and industry in China are lagging behind those of developed countries. Internationally, the study of mine environments entered the initial stage in the early 1970s. In 1972, the United Nations Conference on the Human Environment adopted the Stockholm Declaration, which states that global environmental protection is an important issue of common concern to the international community. Since the 26th International Geological Congress held in Paris, France, in 1980, scholars ended their mine environment research and focused instead on environmental change in the scope of geological research, which was a milestone in the interdisciplinary study of environment and geology [74,75,76,77].

To control environmental pollution and ecological destruction, ensure the rational use of natural resources, and protect and improve the living environment of human beings, many developed countries pay attention to improving environmental protection management legislation in mining through the formulation of laws and regulations to ensure environmental management and protection. Several countries have relatively rich experience in mine environmental management. Mine environmental restoration and management in countries with developed mining such as the United States of America, Germany, Australia, and South Africa began in the early 1970s. Relevant systems for mine environmental protection and restoration and environmental impact assessment were implemented to clarify the responsibilities and obligations of all parties in the provisions of laws and regulations to control the adverse impacts of mining activities on the environment. In 1986, China promulgated the Mineral Resources Law, in which the provisions on environmental protection and pollution prevention during mineral resources exploitation are discussed. After this, the scope of research gradually extended to mine geological disasters, water and air pollution, and other aspects. In the early 1990s, large-scale development led to many scholars becoming aware of the seriousness of mountain forest destruction, soil erosion, river siltation, water quality deterioration, and other problems in mining areas. The prevention and control of geological mining disasters are closely related to production safety and the restoration and countermeasures to address related environmental problems such as water inrush, karst collapse, seawater intrusion, and rocky desertification induced by mine dewatering. However, due to the lack of specialized technical theory regarding mine environmental prevention and control, those in relevant fields, such as highway, railway, and water conservancy, can provide their experience with geological disaster prevention and municipal construction. In the process of environmental restoration and management required as a result of mining, special mine environmental protection management regulations, technical guidance, and management methods are urgently required with mine resources, environmental protection, and water and soil conservation laws.

In recent years, China has launched a large-scale survey of land and resources; mine environment surveys at the regional scale have also gradually begun. The results of comprehensive surveys and assessments of the mine environments in key areas of the country have provided detailed data support for the study of mine environments in China. At this stage, Chinese scholars have gradually begun to study material migration and energy conversion in mines and the surrounding environment, and have discussed the value of the scientific, rational, and green development of mineral resources and the sustainable development of mine environments. The requirements, means, and technical methods of mine environmental investigation have gradually been clarified. Mine environmental restoration and control projects have focused on many fields, such as subsidence pit backfilling, dump slope prevention and control, mine water treatment, mine aquifer restoration, etc. In the era of network “highways”, China has rapidly established a national mine environment monitoring network for real-time information monitoring, and has established and improved local governments’ systems for supervising mine environments. During this stage of rapid development, Chinese scholars have conducted many studies in the field of mine environments, such as the comprehensive classification of mine environmental problems, the evaluation of the environmental impact of mines in different regions of the country, and the characteristics of the comprehensive effects of mines on their environment.

Here, we describe the theoretical and practical results obtained from the studies of the environmental problems caused by mines, investigation technology and methods, evaluation of mine environmental system status, systematic restoration and treatment of mine environments, suitability evaluation of mine land resources after treatment, research and development of dynamic monitoring and early warning technology for mine environments, research and development of mine information management systems, and the soft science on mine environments [78,79,80,81,82,83,84,85,86].

### 3.1. Current Mine Environment Situation and Problems

According to the nature, manifestation, and environmental impact of mine environment problems throughout the life cycle of mines, we collected many studies from the literature; analyzed and sorted the characteristics of different problems after disturbances to a physical, chemical, or biological environmental aspect; and classified the environmental problems as follows [87,88,89,90,91,92,93,94,95].

#### 3.1.1. Environmental Imbalance between Rock and Soil

The problem of environmental imbalance between rocks and soil mass is a main characteristic of areas disturbed by mining. For example, underground or open-pit mining disturbs the mechanical balance of rock and earth mass, resulting in changes in the stress balance between the rock and earth mass within and around the ore body. For example, underground mining causes the deformation and displacement of underground rock and earth mass, and open-pit mining causes the instability of the pit slope. In addition, the land subsidence caused by pumping, secondary geological mine disasters, and slope stability problems experienced by various solid waste dump carriers are ultimately manifested as imbalances in and destruction of the geotechnical mine environment.

#### 3.1.2. Water Environment Imbalances

The problem of water environment imbalance refers to the environmental problems resulting from the disturbance of the water environment around the mine, such as seepage. Some of these problems are the decline in groundwater level, which is caused by changes in the flow field; the leaching of solid waste, which causes surface water pollution; and the pollution of surface water and groundwater, which is caused by production wastewater. Due to changes in the physical and chemical properties of land and the loss of land function caused by the rise in water level owing to mining wastewater and in closed mines, the water chemical field becomes imbalanced in the soil, which also indicates imbalances in the water environment. Another example is the intrusion of seawater into groundwater caused by mining in coastal areas, which directly leads to changes in the hydrochemical field of groundwater.

#### 3.1.3. Atmospheric Environment Imbalances

The annual emission of industrial waste gas from coal mines in China is 395.43 billion cubic meters and that of harmful substances is 731,300, which includes toxic and harmful gases such as smoke, sulfur dioxide, nitrogen oxides, and carbon monoxide. The atmospheric environment has a certain self-purification capacity, but long-term uncontrolled pollution causes atmospheric disturbances, such as the greenhouse effect, acid rain, and so on. The two main imbalances in the atmospheric environment are (1) changes in atmospheric temperature, e.g., the spontaneous combustion of coal seam and gangue releases a large amount of heat, which disturbs the atmospheric temperature field and is an important factor causing climate change in mines; (2) changes in atmospheric composition: waste gas, dust, and waste residue particles (PM2.5) produced by mine operations enter the atmospheric environment through weathering dust under long-term exposure and cause changes in the composition of the air.

#### 3.1.4. Ecological Environment Imbalances

Imbalances in the ecological environment of mines are closely related to the previous three environmental imbalances. The specific manifestations of ecological imbalances are the destruction and disturbances of the soil and groundwater microbial communities, the destruction of plant communities, and the disturbance of benthic communities. After the groundwater seepage field is disturbed and destroyed in the mining process, land desertification occurs on the surface of mining areas. Soil and water losses are caused by the combined action of the destruction of the surface soil in the mining area, the cutting of ground vegetation, and excessive slope surface deformation. In addition, due to the influence of weathered dust, the vegetation community in the mining area is markedly different from the normal vegetation community in terms of morphology and characteristics. For example, under the influence of coal mining dust, the color and growth morphology of some herbaceous vegetation in the Wuda mining area are substantially different from those in other areas.

### 3.2. Environmental Problems in Chinese Mines

China has a vast territory and large differences in natural environments, so the environmental problems caused by mining development show region-specific characteristics and impacts. The analysis of the distribution of mining-related environmental problems is beneficial to understanding the relationship between these environmental problems and factors such as resource distribution, geological environment background, topography and geomorphology, hydrogeological conditions, geological structure, and deposit burial conditions. This information can help guide the study of mine environmental problems, the evaluation of mine environmental status, the prediction of the evolution of these problems, the restoration and management of mine environments, the dynamic monitoring and early warning of mine environments, the mine environmental information system as well as laws and regulations, and the supervision and management of mine environments [95,96,97,98,99,100,101,102].

#### 3.2.1. Western Sandy Desert and Karst Areas of Loess Plateau in Central and Western Regions

The western aeolian desert area has a fragile ecological environment and poor natural conditions. It is distributed in the northwest and southeast of the Junge Basin, the north foot of Qilian Mountain, and the west foot of Helan Mountain, mainly covering the central and western parts of the Inner Mongolia Autonomous Region, the Ningxia Autonomous Region, northern Shaanxi, northwest Gansu, northwest Qinghai, and most parts of Xinjiang. Representative mining areas in this region include Zhunge, Heishan, Table Mountain, Checkerwell, etc. The area has rich coal resources, thick coal seams, high coal quality, and shallow coal burial; the main coal-bearing strata are lower and middle Jurassic, followed by Carboniferous, Lower Permian, and Upper Triassic. Most of the region has a continental climate, rainfall is rare, the air is dry, surface evaporation is strong, and the annual average wind speed is 3–4 m/s. The surface material is loose and porous, surface water resources are scarce, and most rivers are inland. The imbalances in the water and ecological environments caused by mining development in this region are prominent, as evidenced by land desertification, groundwater level decline, and the destruction of surface vegetation communities.

The karst area of the Loess Plateau in central and western China is typical of surface loess and has a temperate arid-to-semiarid climate. The annual precipitation is generally less than 400 mm. Karst has developed in local areas and is distributed in the west of Taihang Mountain, the east of Helan Mountain, the south of Yinshan Mountain, and the north of Qinling Mountain, mainly covering Shanxi, Shaanxi, Ningxia, Gansu, and the central part of Inner Mongolia. Loess is an aeolian and arid sediment with loose structure that dates to the Quaternary. Representative mining areas include Pingshuo, Datong, Xishan, Dongsheng, and Shendong. The main coal-bearing strata are Carboniferous–Permian. The loess landform considerably impacts mine environments, water resources are relatively scarce, and the ecological environment is fragile. In this region, mining development has caused serious environmental imbalances in rock, soil mass, water, and the ecological environment. For example, mining has caused frequent secondary geological disasters in mines, spring water interruption, serious soil and water loss, and low vegetation coverage rates; here, restoration of the mine environment is difficult.

#### 3.2.2. Eastern China High Groundwater Level Area and North China Plain Area

The high groundwater level area in East China is located in the east and southeast of the North China Plain, with high vegetation coverage and abundant surface water and groundwater resources. The climate is temperate humid–semi-humid in Shandong, Jiangsu, Huaibei, and other regions. The coal-bearing strata are Paleozoic Carboniferous-Permian. The representative mining areas are Yanzhou, Jining, Xinwen, Feicheng, and Huainan. The imbalances in the rock, soil, and water environments caused by mining development in this area are prominent. Typical mine environmental problems in Huaibei, Xuzhou, Yanzhou, Zaozhuang, and other coal mining areas with high groundwater levels include perennial or seasonal surface water accumulation and land subsidence.

The North China Plain area mainly includes the Huanghuaihai Plain area, with low mountains and plains as the main terrain, and abundant surface water and groundwater resources. In the typical North China-type coalfield areas, the main coal-bearing strata are Permian coal seams, followed by Carboniferous; Jurassic coal seams are distributed in a few areas. The representative mines include Fengfeng, Xingtai, Liujiang, and Yima. The imbalances in the rock, soil, and water environments caused by mining in this area are prominent and include mining subsidence, groundwater level decline, land occupation, leaching pollution from solid waste stacks, and land salinization.

#### 3.2.3. Karst Area in Southwest China and Middle and Low Mountains and Hilly Areas in South China

The typical environmental problem caused by mining in southwest China is the karst collapse caused by the drainage of groundwater in karst water-filled deposits, which has occurred in Guizhou, Yunnan, Sichuan, and the northern part of Guangxi, among others. Karst areas are located in the Qinba Mountains, Sichuan Basin, Central Yunnan, the Southwest Sichuan Plateau, and Yunnan–Guizhou in Western Hubei. The strata are predominantly Late Permian Longtan–Changxing, with complete coal types and the coexistence of superior and inferior coal. The surface water system and groundwater resources are abundant, and disturbances and destruction of aquifers have widely occurred as they are easily destroyed. The imbalances in the rock, soil, and water environments caused by mining development in this area are prominent, including ground collapse and ground fissures. Land occupation and destruction are widely distributed, and the main land types that have been destroyed are grassland and woodland.

The middle and low mountains and hilly areas in south China are mainly distributed in the central and eastern parts of Hunan, northern Jiangxi, northern Guangdong, and southern Fujian. The terrain is dominated by middle and low mountains and hills. The climate is subtropical–tropical and warm, with abundant precipitation, a developed surface water system, abundant groundwater resources, and high vegetation coverage. The coal-bearing strata are Lower Carboniferous, Lower Permian, and Upper Permian. The imbalances in the rock, soil, and water environments caused by mining in this area are prominent, involving soil erosion, ground collapse, groundwater overexploitation, land occupation, pollution caused by solid waste stacking, secondary geological disasters in mines, seawater intrusion in coastal areas, etc.

### 3.3. Positive and Negative Effects of Mine Environments

The impacts caused by mining activities on the mine environment can be divided into positive and negative, both of which simultaneously occur and are associated with each other. Some studies have aimed to determine the positive effect of the resources in mines, to identify advantages and avoid disadvantages, and comprehensively develop and use the resources, which is an important point supporting the extension of the industrial chain of mineral development. The studies on the negative effects of the mine environment have aimed to find methods of mine environment restoration and treatment to reduce the negative effects of mining activities on the environment [103,104,105,106,107,108,109,110,111,112,113,114].

#### 3.3.1. Negative Effects of Mining

**Figure 8:**
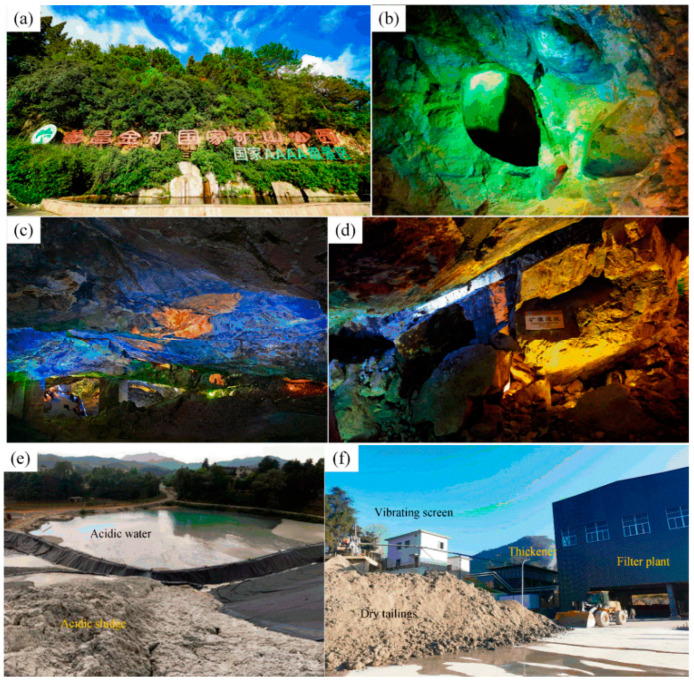
Construction of green mine at Suichang gold mine: (**a**) exterior view of Suichang National Mine Park; gold grottoes of (**b**) Tang Dynasty and (**c**) Song Dynasty; (**d**) mining disaster sites from Ming Dynasty; (**e**) treatment of acid water in old goaf; (**f**) comprehensive use of total dehydration of tailings (Shuai Li [114]).

The negative effects on the mine environment occur through the processes of mineral resources exploration, mining, processing, transportation, and pit closure. Often, many different environmental problems overlap and cause direct or indirect, short- or long-term adverse effects on and damage to the mine environment, which can be divided into four categories in terms of the environment affected: rock and soil, water, atmospheric, and ecological environments (Figure 8).

(1) Negative effects on rock and soil environment

The negative environmental effect on rock and soil mass by mining refers to the disturbances of the balance of the rock and soil environment. Mining changes the balance of material and energy flow and stress; the original surface and the surface of the lithosphere are uncovered, resulting in negative environmental effects. For example, underground mining causes the destruction of the mechanical balance of rock and soil mass, which leads to the deformation and destruction of the surface rock and soil mass structure, evidenced by mining subsidence, secondary geological disasters in mines, ground fissures, land occupation by solid waste piles and open pits, etc., which ultimately lead to the degradation of land functions and the reduction in available land resources.

(2) Negative effects on water environment

The negative effects of mines on the water environment include the physical, chemical, and chemical aspects of surface water and groundwater environments. The comprehensive results of biological disturbances involve the functional disorder of the occurrence, supply, runoff, and discharge of water; changes in the temperature; the disturbance and destruction of microbial communities; the reduction in water resources; and the deterioration of surface water and groundwater quality. The physical disturbances caused by mining include groundwater level decline, caused by mine drainage and mine water inrush, as well as the disturbance of groundwater temperature. Solid waste leaching, liquid waste pollution, groundwater cross-layer pollution, secondary pollution of mine drainage, groundwater level rise in closed mines, and seawater intrusion are the main mine environmental problems that negatively affect the chemical parameters of the water environment. These changes can directly lead to the aggravation of the negative effects on mine environment, and indirectly accelerate the destruction of the environmental balance of rock and soil mass in mines. These environmental effects can seriously threaten human health and the living environment.

(3) Negative effect on atmospheric environment

The negative effects of mining on the atmosphere include changes to air temperature, humidity, wind speed, air pressure, and degradation; changes to physical properties such as water and radiation; and changes to the proportion of gas components, such as increases in harmful gas concentrations. Among them, the disturbance of physical properties is mainly caused by solid waste dust and spontaneous gangue combustion; chemical properties are mainly affected by the toxic and harmful gases produced by spontaneous gangue and coal seam combustion, as well as industrial waste gas. The negative effects on the atmospheric environment by mining are manifested in increases in PM2.5 concentration and acid rain in mining areas. The negative effects on the atmospheric environment are harmful to human health, examples being the London smog event in 1952 and the Los Angeles photochemical smog event in 1955.

(4) Negative effects on ecological environment

The negative effects of mining on the ecological environment include the local destruction of vegetation and soil microbial communities and imbalances in the ecological environment, such as disturbances of groundwater microbial and benthic communities. These lead to changes in the structure of the ecological environment and the decline or loss of function, which is caused by the independent or superimposed negative effects of the geotechnical, water, and atmospheric environments. For example, land desertification, soil erosion, swamping of lowland in mining areas, vegetation degradation, and eutrophication of water bodies in mining areas are caused by spontaneous gangue combustion, land occupation by solid waste stacks, mining subsidence, and solid waste dust.

#### 3.3.2. Positive Effects of Mining

The positive effects of mining on the environment include impacts on space, facilities, associated energy, and places that can be reused or sustainably developed and used in the mining environment and can create certain economic, social, or environmental value. The positive effects of the exploited mine environment can be divided into three categories: space, energy, and comprehensive effects.

(1) Positive effect on mine space

The space around the mine is positively affected because these spaces can be exploited and used after engineering reinforcement, treatment, or restoration after the closure of the mine or the cessation of production. These spaces include the underground areas, underground mine roadways and ventilation roadways, mine buildings, and industrial squares. The space resources can be divided according to their use and nature: storage, scientific research, underground cities, and land resources.

(2) Positive effect on energy

Mining positively affects energy resources in terms of infrastructure remaining in the mine after production or closure, which can continue to produce energy value in the original production location of the mine through transformation, processing, or improvement in mining technology. These include residual, pumped storage power, low-temperature geothermal, coal mine gas power, and photovoltaic power resources.

(3) Comprehensive positive effects of mining

The comprehensive positive effects of mining include the under- and aboveground space resources of the mine, the characteristics of the site, and the ability to transform the area into a non-industrial production site. The development and use options can be divided into popular science education, leisure sports, cultural tourism, and ecological functions, such as mining-based parks, museums, relics, popular science bases, etc. (Figure 8).

## 4. Main Problems Facing Achieving Carbon Neutralization

Given the dual carbon target, a new interpretation is required of the connotations of coal mining and mine ecological restoration. In this regard, China’s mining industry still faces many problems. In this new situation, the objectives of coal mining and mine ecological restoration include the realization of the self-sustainability of the restored ecosystem, positive succession to achieve a new ecological balance, and the sustainable development of the regional social economy, among others. The basic means to achieve these goals is the combination of self-recovery and manual intervention. Through reviewing the literature, we identified the following existing problems. We must realize that entering a postmining era will involve arduous and complex tasks.

### 4.1. Coal Industry Problems in Carbon Neutralization

#### 4.1.1. Rapid Policy Changes and Lagging Structural Adjustment

At present, the public almost overwhelmingly believes that removing coal from the energy structure is the general trend in future energy development. Under the existing technical conditions, 80% of the global coal production capacity may be lost if the total carbon emission limit is set according to the temperature rise control stipulated in the Paris Agreement. As such, the coal industry will face unprecedented challenges in the future [115,116,117,118,119,120,121,122,123].

(1) Coal-favoring policies should be fully withdrawn. The vision of carbon neutrality involves new requirements for China’s social and economic development activities in the next few decades. In the future, China must choose an efficient, clean, green and low-carbon development path. Low-carbon transformation and new energy substitution are the keys to promoting low-carbon development. Restricted by the adjustment of the energy structure, the proportion of fossil energy, mainly coal, in primary energy consumption is bound to be adjusted during the 14th Five-Year Plan period.
(2) A risk exists for capital invested in the early stage of reaching the goals. The coal industry has received huge capital investment and has a long production cycle, and the policy change risk posed by the carbon neutral vision may lead to huge losses for enterprises. The coal and electricity industries are also facing the same problem. In 2008, small- and medium-sized coal mines in Shanxi province were closed down, merged, and transferred. Although illegal mining and protected resources were impacted, the local economy and downstream enterprises, such as coal and electricity, were also harmed, and some problems remained. To date, these issues have not been properly resolved. During the 13th Five-Year Plan period, the state formulated and implemented the reduction rate of energy consumption per unit GDP in each province and limited the total amount of coal burning in each region, thereby adjusting the coal production capacity. The number of mines that closed in China was 12,000 in 2005, and 3000 mines will be closed in the next 10 years. Eliminating this production capacity is needed, but abandoning advanced production capacity is not worthwhile just because of pressure to reduce coal consumption, as those investors will suffer huge losses.
(3) The survival of the coal industry is facing enormous challenges. The goal of carbon neutrality requires carbon emissions to peak as soon as possible, so traditional mining and coal burning are unsustainable. Coal mining and power generation are the areas with the highest carbon emissions. In recent years, China has eliminated hundreds of millions of tons of production capacity and closed hundreds of inefficient coal-fired power plants. CO_2_ rebalancing is the main method through which China will achieve carbon neutrality, which mainly comes from coal power and heating. In addition, a large amount of CH_4_ is emitted from coal mining and transportation. With the establishment of a carbon emission rights market, the coal industry will face huge cost risks and financial pressures in the future if it cannot be passed on to the downstream industries, or once the new energy technology breakthroughs form cost advantages. The coal industry may not survive.
(4) Coal science and technology research and development (R&D) is facing a major test. Existing technologies, such as green mining and clean use, cannot be used to achieve carbon neutrality. The clean use of coal, once regarded as a future strategy, is still insufficient to meet the commitment to carbon neutrality. At present, supercritical power generation technology almost increases the combustion efficiency to the limit, but it cannot decarbonize and provides limited contribution to carbon emission reduction. Oxygen-enriched combustion or combustion capture related to CCUS is conducive to the decarbonization of coal-fired power plants. Once the transaction cost of carbon rights is higher than the cost of CCUS or the cost of CCUS is markedly reduced and economically feasible, CCUS technology will promote the transformation of coal-fired power generation; otherwise, the scale of coal-fired power will dramatically decline.

To summarize, energy-related policy changes, structural adjustment, and other external factors are intertwined, resulting in problems for the survival of the coal industry and the risk of capital stranding.

#### 4.1.2. Standards and Norms Do Not Match, and Regulatory Requirements Are Unsound

In China, the standards and specifications do not match, and the green mine construction standards still need to be revised to meet the vision of carbon neutrality. Currently, the Code for Green Mine Construction in Coal Industry, promulgated by the Ministry of Natural Resources in 2018, is being implemented. Although it involves environmental protection requirements, such as energy saving, consumption reduction, and emission reduction, it is not fully consistent with the new goal of achieving carbon neutrality. Therefore, the focus of the new standard for the coal industry should be as follows: the accounting boundary of carbon emissions must be clearly defined, technological upgrading must advance, emissions must be reduced, and the ecological environment must be restored to increase carbon sinks.

The regulatory requirements are not perfect, and the carbon emission monitoring and information disclosure requirements of coal enterprises must be standardized. The accuracy of the collected carbon emission information is a prerequisite for judging the progress in the realization of carbon neutrality. However, no dynamic monitoring system exists for carbon emissions with high precision and long time series, and the ability to collect and assess carbon footprints is lacking, which hinders any clarification of the contribution of climate-related risks and carbon reduction. In addition, the disclosure of carbon emissions information is an important basis for judging whether the goal of net zero emissions has been achieved, but public disclosure is not mandatory, so the outside world will not know whether net zero emissions requirements have been met.

#### 4.1.3. Insufficient Technology Research and Development and Imperfect Incentive Mechanism

Technology R&D is insufficient, so the R&D of low-carbon and negative emission dual technologies needs to be strengthened. China has achieved considerable progress in the construction of green mines and the clean use of coal, but a gap remains in the realization of carbon neutrality. Taking CCUS as an example, relevant technologies have been implemented around the world, including CO_2_ flooding, CO_2_ flooding of coal bed methane, etc., mainly through pre- and post-combustion capture and oxygen-enriched combustion, among others. As of 2019, China had implemented 21 CCUS projects, with a cumulative CO_2_ storage capacity of about 2 million tons. Some carbon can be bound in natural systems after removal, but most carbon still needs to be permanently stored deep underground to ensure safety. Only highly mature CCUS technology can achieve negative emission potential; however, at present, the technology is still in its infancy and the cost is high, hindering large-scale application.

The incentive mechanism is imperfect, and the incentive mechanism used for emission reduction and sink increases should be rebuilt in line with the vision of carbon neutrality. At present, the incentive intensity and coverage are still insufficient, and the lack of special incentives for low-carbon and zero-carbon investment in coal enterprises is the future trend in carbon market trading. However, the role of the current carbon market in the coal industry is still limited: the coal industry has not yet been linked to the carbon footprint, and the relevant incentive mechanism design is not based on the carbon footprint of investments or assets. Fundamentally encouraging enthusiasm for low-carbon production of coal enterprises is extremely difficult. Coal accounts for more than half of China’s total primary energy consumption; even without considering the huge contribution of coal power to carbon emissions, a huge gap remains between the coal industry and deep decarbonization. To properly address the deficiencies in the standards and norms, regulatory requirements, governance capabilities, technology research and development, and incentive mechanisms, a coal industry development route must be immediately formulated that is aligned with the vision of carbon neutrality.

### 4.2. Problems with Mine Ecological Restoration

Mine ecological restoration refers to the actions taken to restore and the process of restoring the ecological environment that was damaged by mining to the desired state according to local conditions. The research object should be all the ecological environment problems caused by mining.

The Regulations on the Protection of Mine Geological Environment, revised in 2019, describe the prevention and restoration of ground subsidence, ground fissures, collapses, landslides, aquifer damage, and topographic and geomorphological landscape damage caused by mineral resource exploration and mining activities. Where the mining of mineral resources involves land reclamation, the relevant laws and regulations of the state on land reclamation shall be followed. At present, ecological restoration projects integrate land reclamation and mine geology.

Environmental restoration and treatment have occurred, but water and soil pollution have not yet been adequately treated. Regardless of being called ecological restoration, ecological reconstruction, or land reclamation; regardless of the specific technology or means being used; and regardless of whether it is ultimately restored to the original ecological state of the mine or redesigned and constructed, mining damage must be eliminated and the land value must be reasonably restored to ensure ecological function, with green development as the absolute principle [124,125,126,127,128,129,130,131,132,133].

#### 4.2.1. Problems with Mine Ecological Restoration Supervision

The laws and regulations closely related to mine ecological restoration include the Land Reclamation Regulations, Mine Geological Environment Protection Regulations, and Land Reclamation Regulations Implementation Measures, all of which describe the monitoring and supervision of mine environments.

The relevant requirements include the following. For example, according to Article 23 of the Provisions on the Protection of Mine Geological Environment, the departments in charge of natural resources at or above the county level shall establish a geological mine environment monitoring system within their respective administrative areas, improve the monitoring network, conduct dynamic monitoring of the mine geological environment, and guide and supervise mining rights holders to ensure that the mine geological environment is monitored. The mining rights holder shall regularly report the status of the geological environment of the mine to the competent department of natural resources at the county level where the mine is located, and truthfully submit the monitoring data. The responsible department of natural resources at the county level shall regularly report the collected mine geological environment monitoring data to the relevant department of natural resources at the next level. Article 5 of the Measures for the Implementation of the Regulations on Land Reclamation states that the departments in charge of natural resources at or above the county level shall establish an information management system for land reclamation, use the comprehensive supervision platform for land and resources to dynamically monitor the land reclamation situation, and collect, summarize, analyze and publish data and information in a situational manner on land damage and land reclamation within their respective administrative areas. However, at present, the above operation mechanism has not yet been implemented, resulting in a lack of ecological restoration of mining areas in China.

#### 4.2.2. Misunderstandings in Mine Ecological Restoration Engineering

With the large-scale development of ecological restoration projects, construction teams of varying quality are entering the field of mine ecological restoration. Some engineering construction teams have insufficient understanding of the systematic requirements, integrity, and scientificity of mine ecological restoration; their knowledge reserves are insufficient; and their technical skills are limited. Some people think that ecological restoration projects after mining are earthwork projects that involve digging. For example, they think that covering soil on bare rocks and filling soil in collapsed pits are simple tasks, and that the construction is arbitrary. As a result, many restoration attempts have failed. If soil reconstruction is neglected in the process of filling or covering soil, the water–soil–vegetation will not form a healthy relationship, and the vegetation will not survive due to a lack of water and nutrition. As such, the soil will be exposed again after 1–2 years. If the principle of imitating natural landforms is not scientifically used and is not integrated with the local natural environment during shaping of the dump and slope, the maintenance cost will be high, and the long-term stability will be poor, resulting in serious soil erosion and landscape fragmentation. For example, when an acid gangue hill is treated, fire extinguishing may be inappropriate, and fire prevention measures are often not in place, so the reignition rate is as high as 50%. Mine ecological restoration projects need scientific and technical support.

#### 4.2.3. Difficulties in Popularization and Application of New Technologies

**Figure 9:**
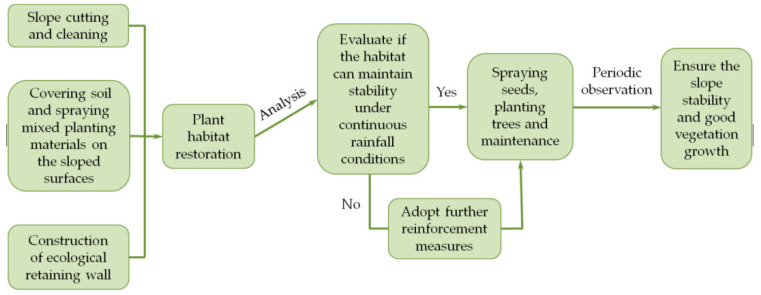
Ecological restoration process of abandoned island mine (Li, X [133]).

After more than 40 years of practice and research on ecological restoration of mining areas in China, researchers have developed a variety of appropriate technologies, but the popularization and application of these new technologies are relatively limited. At present, the ecological restoration of mines in China mostly depends on government investment, especially for older mines, and the social investment is low, so the funds are limited. Some new technologies are more expensive, even though they produce a better repair effect. For example, soil reconstruction technology is less expensive compared to extensive, one-time excavation and filling and layered stripping and staggered backfilling according to the needs of vegetation growth, so construction enterprises are not motivated to use this technology. Another example is the technology used for mining during underground subsidence, because construction occurs when the ground is not stable, so the elevation of subsequent subsidence needs to be considered, which means that the ground is not flat during the project, which does not meet the traditional acceptance requirements. As such, construction enterprises and local governments are unwilling to use this technology logically, which causes a loss of soil resources. Therefore, new technologies must be promoted, requiring managers and builders of ecological restoration projects to change their thinking and achieve breakthroughs in investment and policy (Figure 9).

## 5. Policy Suggestions

China’s future is decarbonization, but not every industry will be required to achieve full carbon neutrality. Coal is still the cornerstone of national energy security; ecological restoration is the foundation of development, with the important responsibility of bottom-up security. As such, blindly abandoning coal is not feasible: green mining and the scientific use of coal are the keys to ensuring the realization of carbon neutrality. In addition, coal carbon emissions mainly come from use, so the carbon sources formed by downstream use such as coal power and the coal chemical industry should not be attributed to the coal industry. However, due to policy bias, structural adjustment, lack of science and technology, and other factors, the future coal industry and ecological modification technology will require considerable alternations and transitions [134,135,136,137,138,139,140,141,142,143].

### 5.1. Proposals for Development of Coal Industry toward Carbon Neutrality

#### 5.1.1. Construction of Carbon Footprint Accounting System for Coal Life Cycle

The whole coal life cycle includes construction, mining, transportation, processing, use, mine closure, ecological restoration, and other stages, all involving the carbon cycle. However, the accounting of the carbon footprint of the whole life cycle of the coal industry should not consider the carbon generated by coal use in downstream industries. In the above stages, except for ecological restoration increasing carbon sinks, the other aspects may increase carbon emissions, for which the carbon footprint can be monitored and assessed. The existing technology can also achieve carbon emission reduction to a certain extent by, for example, using energy-saving equipment, reducing exhaust emissions, and improving coal use. Ensuring that carbon sinks are not reduced has become a problem that the coal industry must face and solve. In addition, a huge amount of CH_4_ is emitted by coal mining in China; thus, if the CBM can be extracted and used before or during coal mining, carbon emissions may be further reduced. Land damage caused by coal mining indirectly causes vegetation damage, soil carbon pool loss, biodiversity reduction, and so on. Other threats, such as spontaneous combustion in coal fields in western China, must also be considered. These concomitant problems can also change the carbon footprint. Therefore, the carbon source and sink effects throughout the whole coal life cycle must be clarified, the interaction mechanisms of the carbon cycle need to be analyzed, and a carbon footprint accounting system must be constructed from the perspective of the whole life cycle of coal, so as to assess or predict the greenhouse gas emissions of simulated coal mining areas in different periods and stages.

#### 5.1.2. Response Hierarchy and Implementation Mechanism

Land degradation balance (LDN) is one of the sustainable development goals of the United Nations for 2030. Neutrality is part of these goals—that is, to achieve “net zero degradation” by means of balance. The response hierarchy of LDN is “avoidance prior to reduction, reduction prior to recovery”. The coal industry can also refer to this standard to achieve carbon neutrality. Therefore, the mechanisms through which carbon neutrality can be achieved in the coal industry include (1) measures to avoid or reduce emissions from the source to promote the realization of near-zero emissions and (2) determining the emitted CO_2_ via the dynamic monitoring of the carbon cycle, predicting the potential emission of CO_2_ in the future, and implementing effective measures to offset the emitted greenhouse gases, forming net zero emissions characterized by carbon balance. Here, we need to further define the conceptual difference between “near zero” and “net zero”. Even if low-carbon technology achieves substantial changes in the future, a “zero emission” of the coal life cycle and its downstream use will be difficult to achieve, but “near zero emission” at the source is possible.

In addition to achieving near-zero emissions, carbon sinks must be expanded through negative emission technologies and ecological restoration to offset the emission of CO_2_.

#### 5.1.3. Agglomeration and Synergy to Increase Carbon Sinks

Agglomeration and synergy: With the increasing pressure to reduce carbon emissions, the quality requirements of coal products will rise, and the market competition will become increasingly fierce. As such, the formation of new standards in the coal industry chain must be promoted, which should extend downstream to increase the added value of the industrial chain. Therefore, an industrial system must be built with the coal industry as the source, and the downstream industrial chains, such as coal power and the coal chemical industry, should gather and connect with each other to promote clean and efficient cogeneration technology, special coal supercritical circulating fluidized beds, and other efficient, clean power generation technologies to increase the proportion of low-carbon raw materials, reduce the carbon footprint of the whole life cycle, and promote the coordinated carbon emission reduction in both up- and downstream industrial chains.

Increase carbon sinks: Emission reduction measures based on natural solutions (such as afforestation) are important to expand ecological carbon sinks, and ecological restoration of mining areas can effectively help with achieving the vision of carbon neutrality in the coal industry. Previously, the ecological restoration of mining areas focused more on site restoration, and the restoration rate was less than a quarter. The main coal mine areas in China are located in the central and western regions where the climate is arid and the ecology is fragile. Ecological restoration in this area is not only costly, but also has little effect.

#### 5.1.4. New Energy Coupling and Adaptation to Carbon Market

New energy coupling: Breaking the bottleneck of carbon emissions of coal will be difficult in the short term, and the cost of emission reduction will not considerably reduce. The problems of low-cost and large-scale storage of renewable energy are problems that still need to be solved, and increasing access to the energy system is difficult. In the future, we will still need to use coal-fired power during peak periods to ensure the basic supply of energy; however, carbon emission reduction is still required and a supporting basis must be provided for the development of new energy. Coupling of coal and new energy will form a new energy structure. An advanced energy system, which integrates wind, solar, water, fire, and storage, is needed to substantially reduce the carbon emissions of coal.

### 5.2. Suggestions for Ecological Restoration and Management

The purpose of ecological restoration of mining areas is not only to restore damaged terrain and greening—ecological restoration of these areas requires a systematic project, which is complex, integrating damage investigation, design planning, and construction. To restore damaged ecology, deeply understanding the connotation of the restoration goal is necessary; at the beginning of planning, the land use, ecological structure, and ecological functions that should be achieved after restoration must be clarified. Therefore, the goal setting for the ecological restoration of mining areas should follow several principles: respect for nature, people-oriented; adjust measures to local conditions, in line with the overall regional planning; safe, efficient, and sustainable use; prioritize ecological and environmental benefits; pay attention to economic benefits; prioritize the restoration of cultivated land, grassland, and woodland; combine end control with source and process control [143,144,145,146,147,148,149,150,151].

#### 5.2.1. Strengthening Basic Research on Ecological Restoration of Mining Areas

The 40 years of experience with the ecological restoration of mining areas have shown that the theory of restoration is far behind the practice of restoration; in many cases, restoration fails because of the lack of scientific restoration, which indicates a decoupling between theory and practice. To repair the damage to the ecological environment caused by mining, many places have spontaneously repaired and used the damaged land and ecological environment. For densely populated areas with rapid economic development, ecological restoration of mining areas is often more quickly promoted. However, the basic theory and repair technology principles still need to be studied in depth to support and promote the development of this field. Faced with the complicated problems of the environmental damage produced by mining, repair means and technologies urgently need to be innovated, enriched, and promoted. We should start with the scientificity, difference, advancement, and economy of technology; enrich the understanding of scientific repair of the environment; and balance the benefits of repair with capital and policy input. Although many kinds of ecological restoration technologies are available, some basic common technologies are often used in ecological restoration, which is also the key to ecological restoration. Water is the source of life, soil is the foundation of life, and plants are the root of life. Therefore, water, soil and plants are the three major elements in ecological restoration. The restoration technologies focused on these three elements form the core of restoration: landform remodeling, soil reconstruction, and vegetation restoration.

#### 5.2.2. Improve and Implement Regulatory Mechanism

As mentioned above, the Regulations on the Protection of Mine Geological Environment and the Measures for the Implementation of Land Reclamation Regulations all describe requirements for the monitoring and supervision of the ecological restoration of mining areas. However, because they involve mining enterprises, the public, restoration, enterprises, and other interests, implementation has not yet occurred. In 2021, the Ministry of Natural Resources planned to complete the verification of damaged patches of abandoned mines across the country, then formulate an ecological restoration plan after the verification and monitor the treatment results every year. It simultaneously plans to strengthen the supervision and management of ecological restoration of production mines in combination with the reform of mining rights, and normalize the annual report system. Some provinces and municipalities are trying to establish a large data platform for the ecological restoration supervision of mining areas to gradually implement the supervision mechanism. If the mechanism operates normally, the old ecological restoration projects will be completed each year, and the number of new projects will not increase or will increase less. This is a feasible method to monitor and supervise ecological mine restoration with the help of a big data platform. Therefore, in the new round of land space and ecological restoration planning, a database must be established from province to county, and the ecological restoration work should be mapped, including the ecological mine restoration work. For example, the large data platform of land and space ecological restoration in Shaanxi Province can be used to monitor and manage the restoration of the geological mine environment in Shaanxi Province.

We require comprehensive information on a unified platform, with a unified portal and unified management, including information on reclamation, the development management, the comprehensive management of land and space, and the restoration of mountain, water, forest, field, lake, and grass systems. An annual reporting system of ecological mine restoration is also required.

In the future, we must focus on improving the reporting and the approval and acceptance system, clarify the responsibilities and time requirements for each link, and formulate corresponding incentive and punishment measures.

#### 5.2.3. Strengthen Regulation and Management and Build Effective Policy System

A theoretical system of mine environment restoration and management mode is described in this paper. From the types of mineral resources, the development of mineral resources methods, mining factors, and mine environmental sensitivity were used to analyze the objects of mine environmental restoration and control layer by layer, and to sort out the main factors controlling the objects of restoration and control. The objectives of restoration and management were divided into disaster elimination and management, land use, and ecological restoration objectives from multiple perspectives. We summarized the mining subsidence, solid waste, and open-pit slope stability problems facing mine environment repair technology. Three sets of repair and treatment modes were constructed, which are suitable for addressing the subsidence, solid waste, and slope stability problems of open-pit mines.

## 6. Conclusions

The development of human society benefits when the environment of the Earth is healthy. When human demands exceed the carrying capacity of the Earth, the ecological environment will collapse, and human beings will go extinct because of the loss of their only home. Building a carbon-neutral society is about preventing this terrible outcome and finding methods to sustain the planet’s ecology forever. The ultimate goal of carbon neutralization is to build a green Earth and a livable home, to achieve harmonious coexistence between human beings and the Earth, which requires building a carbon-neutral society. The Earth is our only home. The carbon emissions caused by human activities have affected the ecosphere. Everyone is responsible for the increase in carbon emissions. As such, restoring the clean atmosphere of the Earth also requires everyone’s efforts. Building a carbon-neutral society is required to save the Earth as well as human civilization. Establishing a carbon-neutral social order and compensating for the damage we have caused to the Earth require the recognition and efforts of everyone, every enterprise, and every country. Carbon neutrality is the basic condition to ensure that the Earth has fresh air, a suitable temperature, vigorous vitality, and clean space.

The key to achieving the goal of carbon neutrality lies in the transformation of the energy structure. To reach the goal of carbon neutrality, carbon-based energy needs to transition to non-carbon-based energy. The transformation to low- and zero-carbon systems will accelerate, the use of fossil energy will gradually change from the main energy source to a back-up energy source, and new, clean energy will gradually become the main energy used. To achieve the strategic goal of carbon neutrality, the arrival of the new energy era must be accelerated. The development of new energy and ecological restoration governance are keys to achieving a carbon-neutral society and building a green and livable Earth. When the whole of human society is included in the carbon-neutral system, we will regain a green Earth and livable home for a long time.

In this study, we collected data and performed an in-depth analysis of the basic and structural characteristics of the development of the coal industry and environmental remediation. We studied and judged the trend in regional economic development and demand growth, closely examined the requirements of China’s development strategy, and discussed the dual goals of carbon peak and carbon neutralization in line with local development trends and economic system characteristics. We must build a livable Earth, promote the green and low-carbon transformation of regional energy, promote high-quality economic development, and ensure the safe supply of energy.

## Figures and Tables

**Table 1:** Some of the large-scale CCUS integration demonstration projects in China.

Project	Capture Mode	Transportation	Storage/Use	Scale (10,000 t/a)
CCS Project of Sinopec Shengli Power Plant	Power plant	Pipeline transportation	EOR	100
	Capture after combustion	Distance: 80 km		
Datang Group CO_2_ Capture and Storage Demonstration	Power plant	Pipeline transportation distance: 50~100 km	EOR or brackish aquifer sequestration	100
	Oxygen-enriched combustion trapping			
Shanxi International Energy Group CCUS Project	Power plant	Pipeline transportation	Not clear	200
	Oxygen-enriched combustion trapping			
Shenhua Ningxia Coal-to-Oil Project	Coal to liquid	Pipeline transportation distance: 200~250 km	Not clear	200
	Capture before combustion			
Huaneng Green Coal Power IGCC Project Phase III	Power plant	Pipeline transportation distance: 50~100 km	EOR or brackish aquifer sequestration	200
	Capture before combustion			
Phase II of Shenhua Ordos Coal-to-Oil Project	Coal to liquid	Pipeline transportation distance: 200~250 km	Saline water layer is sealed	100
	Capture before combustion			
Shenhua Guohua Power Shenmu Power Plant CCS Project	Power plant	Pipeline transportation	EOR or brackish aquifer sequestration	100
	Oxygen-enriched combustion trapping	Distance: 80 km		
China Resources Power Carbon Capture and Storage Integration Demonstration Project	Post- and precombustion capture in power plants and oil refineries	Pipeline transportation	Offshore EOR or brackish aquifer sequestration	100
		Distance: 150 km		

## Data Availability

Not applicable.
